# The Safety and Efficacy of the Continuous Peripheral Nerve Block in Postoperative Analgesia of Pediatric Patients

**DOI:** 10.3389/fmed.2018.00057

**Published:** 2018-03-09

**Authors:** Dušica Simić, Marija Stević, Zorana Stanković, Irena Simić, Siniša Dučić, Ivana Petrov, Miodrag Milenović

**Affiliations:** ^1^Department of Anesthesiology and Intensive Care, University Children’s Hospital, Belgrade, Belgrade, Serbia; ^2^Medical Faculty University of Belgrade, Belgrade, Serbia; ^3^Department of Orthopedic Surgery, University Children’s Hospital, Belgrade, Belgrade, Serbia; ^4^Department of Anesthesiology and Intensive Care, Clinical Center of Serbia, Belgrade, Serbia

**Keywords:** pediatric anesthesia, continuous peripheral nerve block, postoperative analgesia, pain management, perineural catheters

## Abstract

Postoperative analgesia is imperative in the youngest patients. Pain, especially if experienced during childhood, has numerous adverse effects—from psychological, through complications of the underlying disease (prolonged treatment, hospital stay, and increased costs of the treatment) to an increase in the incidence of death due to the onset of the systemic inflammatory response. Peripheral blocks provide analgesia for 12–16 h, and are safer due to rare side effects that are easier to treat. The continuous peripheral block (CPNB) has been increasingly used in recent years for complete and prolonged analgesia of pediatric patients, as well as a part of multidisciplinary treatment of complex regional pain syndrome. It has been shown that outpatient CPNB reduces the need for parenteral administration of opioid analgetics. It has also been proved that this technique can be used in pediatric patients in home conditions. Safety of CPNB is based on the increasing use of ultrasound as well as on the introduction of single enantiomers local anesthetics (ropivacaine and levobupivacaine) in lower concentrations. It is possible to discharge patient home with catheter, but it is necessary to provide adequate education for staff, patients, and parents, as well as to have dedicated anesthesiology team. Postoperative period without major pain raises the morale of the child, parents. and medical staff.

## Mini Review

The pain, according to the new definition, is a disturbing experience associated with existing or potential tissue damage, with a sensory, emotional, cognitive, and social component (1). Postoperative analgesia is imperative in the youngest patients. Pain, especially experienced during childhood, has numerous adverse effects—from psychological, through complications of the underlying disease (prolonged treatment, hospital stay and increased costs of the treatment) to an increase in the incidence of death due to the onset of the systemic inflammatory response (2). It is clear that all those who deal with child health care have a moral obligation to prevent and adequately cure their pain.

Regardless of immaturity, the child can feel pain since birth (3). The pain sensitivity is greater in younger children and so analgesia should be provided to them during, before and after the surgery since birth. Repetition of painful procedures determines the threshold for pain for the whole life (4). Inadequate treatment of acute pain is one of the important prerequisites for the development of chronic pain.

The goal of analgesia in the postoperative period is to reduce or eliminate pain with minimal additional harmful effects and overall treatment costs. Adequate postoperative analgesia, especially during the first 48 h, reduces the stress response of the organism to the surgical procedure, thereby affecting endocrine, metabolic, and inflammatory changes. This reduces the incidence of postoperative complications and improves the outcome of surgical treatment (4–8).

Single shot peripheral regional blocks provide analgesia for 12–16 h, almost the same length as central blocks (9). Peripheral blocks are safer due to rare side effects that are easier to treat and their use has increased significantly over the last two decades (10). The continuous peripheral block (CPNB) has been increasingly used in recent years for complete and prolonged analgesia of pediatric patients, as well as a part of multidisciplinary treatment of complex regional pain syndrome (CRPS) or epidermolysis bullosa (11).

It has been proven that outpatient CPNB reduces the need for parenteral administration of opioid analgesics (12). It has also been proven that this technique can be used in pediatric patients in home conditions (13, 14). Patients can be released home even with a residual motor block, after prescribed additional oral analgesic therapy and after they received verbal and written instructions regarding the use of CPNB and the identification of possible complications (muscle weakness, less feeling for hot or sharp objects…) (13). There is currently no publication on the wound catheter technique in pediatric patients (11).

There are still not enough prospective studies to confirm the efficacy and safety of CPNB technique and the existing studies have numerous limitations. Comparison of studies is difficult due to differences in the definition of side effects.

Safety of CPNB is based on the increasing use of ultrasound (US) as well as on the introduction of single enantiomers local anesthetics (LA) (ropivacaine and levobupivacaine) in lower concentrations. Originally, bupivacaine had a primacy, while today ropivacaine (0.1–0.2% by infusion, average 0.25 mg/kg/h) is mainly used because of its lower toxicity (9, 15, 16). This LA also provides a better differential block (sensory block without motor nerve paresis) (17). It can also be used for a patient controlled administration (0.2% ropivacaine 0.02 ml/kg/h, bolus 0.1 ml/kg, lockout interval 30 min) (18). The risk of LA toxicity to muscle tissue is increased in infants, so it is advised to use the lowest possible doses and concentrations of LA (11, 19).

However, if catheter efficiency is suspected in the immediate postoperative period, it is recommended to perform a test bolus dose of 3 ml of lidocaine 1.5% with epinephrine 1:200,000 (15). If tachicardia appears, the catheter is placed intravascularly.

Peripheral regional block in children has faster onset but short duration. In children under 1 year of age, nerve fibers are thinner, myelination is scarce, and Ranvier’s nodes are closer. The volume of distribution is higher (20, 21), clearance is smaller (22), and the free drug fraction (unbounded for proteins) is higher (20) so the doses are almost the same as in adults. Cytochrome CYP1A2 on which ropivacaine is metabolized matures around the age of 4–7 years old, and CYP3A4/7 on which levobupivacaine is metabolized matures at the end of the first year (23).

Catheters are placed in sterile conditions with the help of a nerve stimulator (4.6%), ultrasound (30.2%), or a combination of these two techniques (62.9%) (15). Placement of perineural catheters under the control of US is becoming more and more frequent and has an increasingly wider use nowadays. In the study of Walker et al. (9), the ultrasound has advantages (in up to 90% of the cases depending on the type of block). Advantages of using US are reflected in the fact that it is possible to monitor the path of anatomical structures to achieve a safe orientation. The latest US devices allow visualization of the needle itself and in that way they ensure the best position of the needle in relation to the anatomical structures, reduce the risk of nerve injury and surrounding structures. US enables monitoring of the distribution of LA, preventing intravascular injection and optimizes the amount of LA which reduces the risk of toxic reactions. The catheter was usually placed under general anesthesia [in 92.9% of patients by Visoiu et al. (15) and in 98.9% of cases by Gurnaney et al. (12)].

Patient satisfaction is a very important indicator of the quality of treatment and higher pain control satisfaction score (PCSS) is an indicator of better patient care. Pediatric patients have expressed satisfaction with the popliteal CPNB (24). For the first time, Visoiu et al. evaluated pediatric patient satisfaction with analgesic therapy using PCSS and 91.4% of patients were very satisfied (8–10 out of 10) (15). They reported home PCSS for parents (9–10 out of 10) and for medical staff (9–10 out of 10). In Visoiu et al. study, more patients reported pain at home than during the Postoperative Ambulatory Care Unit (PACU) stays. Pain scores were lower in the PACU and on postoperative day 0 than on postoperative day 1 and the following days (15).

In a study by Visoiu M et al., 31.4% of patients did not have pain and did not receive any additional analgesics during their stay in PACU. After the release from the hospital, 25% of the patients did not have any pain at home, although 97% of patients received at least 1 dose of opioids (15). According to Dadure et al. about 60% of patients received at least 1 additional dose of oral analgesics (25). Study of Ganesh et al. showed that about 56% of children received opioid during the first eight postoperative days (13). The average time for the first dose of opioids in Gurnaney’s study was 16 h (12). 60% of their patients needed an opioid within the first 8 h 40% of which received the opioid already in the recovery room (12). The incidence of patients needing opioid analgesia increased to about 74% by 48 h with about 26% of the patients not requiring any opioid analgesics (11). The reason for the frequent use of opioids could have been due to the preference of lower concentration and infusion rate of LA to avoid motor block (recommended 0.4 mg/kg/h maximum infusion rate for ropivacaine). Another reason could be that multiple nerves need to be blocked, to provide complete sensory block after certain procedures.

Continuous peripheral block does not exclude the additional use of opioids (13). In the PACU surgical analgesia is usually achieved by CPNB but some other pains (tourniquet) or reasons to be restless remains (anxiety, due to the absence of the parents, emergence delirium associated with sevoflurane, etc.). Postoperative use of opioids in home conditions is likely to occur because parents are advised to give the prescribed medication (as needed) to the children with low intensity pain or before going to sleep (15).

Perineural catheter technique is used in chronic pain. The management of patient with CRPS is integrated in a multidisciplinar approach associating pain management with physiotherapy treatment and psychological management. The keys to success are active physiotherapy treatment and restoration of normal limb movement to which CPNB may contribute. Recurrent CRPS remains a therapeutic challenge in pediatric patients. Dadure et al (11), reported that a 4-day CPNB after an initial Bier block is effective against intractable and recurrent CRPS in 13 children, leading to pain reduction, physiotherapy facilitation, and functional rehabilitation. In this study, all children were able to move about easily after the initial 24-h period and continued the treatment at home using infusion pumps.

Complications are rare and minor, mainly mechanical (accidental catheter withdrawal, dislodgement, or occlusion), and nausea and vomiting (11). So far, the largest study on the safety of perineural catheters use in children on over 2,000 set up catheters in children demonstrated a low degree of complication, which is correlated with the percentage of complications in the adult population (15).

In the study by Visoiu et al., 14.4% of patients had subsequent catheter-related complications, mainly minimal catheter leakage that did not affect the analgesic effect (15). Dadure et al. reported 20.1% of mechanical problems associated with catheter (mainly leakage and dislodgement) (25). In a study by Ganesh et al. (13), the catheter was accidentally withdrawn in 40.5% of patients. Despite the use of good fixation, Walker et al. noticed the occurrence of subcutaneous catheter migration that may result in secondary block failure (9). Gurnaney et al. had 4.2% catheter complications, 1.9% catheter failure, and only 0.07% of local inflammation (12). There were no differences in the risks and complications in inpatients and outpatients. It is necessary to improve the technique of catheters positioning and fixing (12, 24). Some authors recommend the use of a small drop of Dermabond (Ethicon, Raleigh, NC, USA) at the insertion site (12, 15, 16) to prevent leaking.

Visoiu et al. reported that 28% of patients had postoperative nausea/vomiting and/or itching (15). The technique did not work in only 6.9% of cases, but even this small percentage is unacceptable for patients and medical staff. In the study by Dadure et al. 14.7% patients had nausea/vomiting and only 1.5% urinary retention and 0.9% pruritus (24). Gable et al. reported postoperative nausea and vomiting in 5.9% of patients (14).

In the study by Walkers et al. (9), there were no permanent neurological complications of deep infection or local anesthetic toxicity, but most patients were older than 10 years. There were no permanent neurological complications of deep infection or LA toxicity in other studies as well (10, 15). The most common local complications are rare: local inflammation at the site of catheter placement (redness, swelling, or pain) and abscess at the catheter insertion site. Ecoffey noticed only superficial infections or blood vessel puncture (10). Studies have shown that perineural catheters infections are a rare occurrence and that the incidence is in correlation with the time that has passed since the catheter is placed (9, 12, 13). It is considered that the perineural catheters should be removed 3 days after the placement which reduces complications to a minimum (15), except in cases where the benefits to the patient overcomes the clinical risk of infection.

Many complications (e.g., paresthesia) are difficult to diagnose in infants and nonverbal children who cannot describe their symptoms accurately. Nevertheless, in a study by Polaner et al. (26) the incidence of serious complications that was detected in prospectively acquired unselected population was extremely small, and no sequelae lasting >3 months were reported in close to 15,000 regional anesthetics. There were no serious complications such as persistent neurological deficit. In these instances, we must rely on confidence intervals to provide an upper limit of possible incidence rates (for example, although there was no mortality reported in 9,156 neuraxial blocks, a mortality of 0–3.3: 10,000 is still consistent) (26).

Krane and Polaner believe that it cannot be determined whether the rare symptoms of LA (e.g., tinnitus) are objective or only placebo responses in children who are told to pay attention to these symptoms of LA toxicity (27).

Absolute contraindications for placement of perineural catheters are: allergy to local anesthetic and infection at the site of planned puncture. Relative contraindications are sepsis, prolonged PT and PTT, heart failure, neurological diseases, and patient refusal. Due to the small number of contraindications and improvements in the clinical, economic, and humanistic approach, perineural catheters are used more often nowadays (15).

## Conclusion

Regional anesthesia is commonly used in addition to general anesthesia to provide adequate postoperative analgesia and better comfort. It provides sufficient analgesia and better comfort and it is rarely performed in a wake state. Postoperative course without the significant pain raises the morale of the child, parents, and medical staff. The surgeon as well as the anesthesiologist is pleased to see a peaceful, alert and cooperative child in the immediate postoperative period. From an ethical point of view, it is not justifiable to allow the child to suffer pain, when simple and safe techniques of regional anesthesia are easily complementing or replacing conventional-general anesthesia. The goal of a physician should always be to minimize the psychological and physical trauma of the patient, regardless of how young and immature the child is. Hospital stay will be forever remembered as a traumatic experience if pain is not adequately treated. Therefore, proper care of pain is of great importance.

The use of any technique of regional anesthesia depends on the estimated risk/benefit ratio. No published study reported sustained neurological complications or serious side effects after use of CPNB. It is possible to discharge patient home with the catheter, but it is necessary to provide adequate education for staff, patients, and parents, as well as to have dedicated anesthesiology team. It is extremely important to organize adequate monitoring of these patients by phone calls and visits by trained medical workers. Regardless of the numerous ethical and security problems in the design of pediatric studies, more prospective studies are needed to provide adequate evidence.

## Author Contributions

DS: conceptualization, investigation, project administration, visualization, and wrote the manuscript. MS: investigation, visualization, literature review and wrote the manuscript. ZS: literature review, providing regional anesthesia, visualization and investigation. IS: placing perineural catheters, providing regional anesthesia, visualization, and investigation. SD: investigation and supervision. IP: providing regional anesthesia, language supervision and made a final version of manuscript. MM: visualization, analysis of literature, and investigation.

## Conflict of Interest Statement

The authors declare that the research was conducted in the absence of any commercial or financial relationships that could be construed as a potential conflict of interest.
